# RNA Sequencing and Spatial Transcriptomics in Traumatic Spinal Cord Injury (Review)

**DOI:** 10.17691/stm2023.15.6.08

**Published:** 2023-12-27

**Authors:** Yu.A. Chelyshev, I.L. Ermolin

**Affiliations:** MD, DSc, Professor, Department of Histology; Kazan Federal University, 18 Kremlyovskaya St., Kazan, the Republic of Tatarstan, 420008, Russia; DSc, Professor, Department of Histology with Cytology and Embryology; Privolzhsky Research Medical University, 10/1 Minin and Pozharsky Square, Nizhny Novgorod, 603005, Russia

**Keywords:** spinal cord injury, RNA sequencing, spatial transcriptomics

## Abstract

In order to understand the fundamental mechanisms of the spinal cord functioning, it is necessary to reveal a complete set of cell types and their populations, which can be identified by the unique combination of their features. The technologies of single-cell and single-nucleus RNA sequencing serve as effective tools for determining the role of various types of cells in normal and pathological reactions in the spinal cord. Spatial transcriptomics combines these technologies with the methods of obtaining and saving spatial information about cells in the tissue, which allows one to localize more precisely the injured area, characterize in detail the tissue compartments in the specific anatomical region, and analyze the pathological picture at the cellular and molecular level.

Atlases of development of RNA-sequencing technologies and spatial transcriptomics created on the basis of the data from single-cell and single-nucleus RNA sequencing open great opportunities for new perspective concepts concerning the mechanisms of rearranging neural connections and restoration of sensorimotor functions in traumatic spine injury. The transcriptomes obtained were a powerful resource for detecting new functions of the nervous tissue cells. To establish therapeutic targets, the detected molecular diversity in neurons of various types enables tracing and comparing their susceptibility and regenerative potential. Determination of causes of selective cell susceptibility in spinal cord injury needs comprehensive information on the specificity of human cell populations in comparison with the known data obtained on the experimental models.

In the present review, we have summarized advances in identification and study of cell characteristics in a traumatized spinal cord based on transcription profiling at a single-cell or single-nucleus level.

## Introduction

Sensory information processing and locomotor reactions in the spinal cord are controlled by neural networks incorporating cells of many types [1–3]. To establish cellular correlates of behavioral reactions, it is important to have knowledge of specific types of cells forming the neural network. The solution of this task became possible owing to the development and application of RNA sequencing (RNA-seq) technologies of a single cell (scRNA-seq) or a single nucleus (snRNA-seq), an effective tool for understanding the role of various types of cells in normal and pathological reactions in the nervous system. These technologies make it possible to present the distribution of the specific type of cells by visualizing gene expression in the context of a tissue structure (spatial transcriptomics).

The investigation of Cahoy et al. [4] served as a conceptual and technological breakthrough in neurosciences as the first classification of CNS cell populations based on transcriptome profiles created using the results of researches applying microchip technologies. The transcriptomes obtained were a powerful tool for the understanding of new functions of the nervous tissue cells. Later, this classification was specified and complemented using scRNA-seq/ snRNA-seq technologies and spatial transcriptomics.

An understanding of the fundamental mechanisms of spinal cord functioning requires the detection of a complete set of cell types and their populations, which can be identified by the unique characteristic combination. A specific type of the spinal cells is characterized by many parameters such as localization, structure, cytogenesis, electrophysiological properties, involvement in network formation, patterns of gene expression, and involvement in behavioral reactions [5]. It is not obligatory to have a complete set of characteristic features for identification of the specific cell type, but their combination underlies cell identity.

During sample preparation for scRNA-seq, enzymatic cleavage results in the destruction of synaptic structures and neuron death. snRNA-seq, as compared to scRNA-seq, can minimize the generation of pseudocellular structures induced by enzymatic hydrolysis and mechanical damage. Besides, snRNA-seq is supposed to collect information on introns and intergenic regions and characterize rarer types of cells. Although snRNA-seq enables one to obtain more complete information on the cell types, the scRNA-seq technology appeared to be more suitable for the description of some types of cells, for example, immune cells [6, 7].

The methodology of RNA sequencing includes the following stages: 1) dissociation of tissue and obtaining separate cells; 2) amplification of nucleic acids; 3) high-throughput sequencing; 4) data analysis. The detailed characteristic and traps and pitfalls of each stage are considered in the works [8–13].

Spatial transcriptomics combines scRNA-seq with the methods of obtaining and saving spatial information in the tissue. This approach allows one to precisely localize the injured region and to consider its pathogenesis at the cell level [14]. If compared to the standard scRNA-seq technology, the resolution and effectiveness of gene detection in the process of spatial transcriptomics are insufficient, but bioinformatics algorithms give the possibility to achieve the desired approximation.

Methods of spatial transcriptomics are used to create the maps (atlases) of gene expression in any tissue compartments. Such harmonized atlases of cell types and their distribution have already been created for a mouse spinal cord [15–17]. In these atlases, a large panel of markers is engaged for exploring the cells of different types both *in vivo* and *in vitro*, the supposed embryonic lines for each type of cells are presented, and computational resources for classification of spinal cord cells based on transcriptomics are also mobilized, allowing the researchers to readily interact and analyze the data on specific cells of the spinal cord [17]. These atlases can serve as a universal nomenclature both of cell types and a set of molecular markers, which together characterize in great detail tissue compartments in a specific anatomical region.

The scRNA-seq/snRNA-seq technologies play an important role for establishing a standard set of cell types in the spinal cord and understanding molecular mechanisms of pathological shifts in this organ. Such technologies enable one to perform screening of the differentially expressed genes at various times after spinal cord trauma (SCT). Thus, in contusion SCT at Th8 level in the rat, the number of genes with significantly changed activity was 944, 1362, and 1421 on days 1, 4, and 7, respectively, after the traumatic event [18]. Using scRNA-seq, hundreds of molecularly diverse types of cells have been revealed in the mouse and human nervous system, whose function is determined by a differential activity of genes and topography of the specific cell types [16, 19, 20]. Application of the sequencing technology together with spatial transcriptomics makes it possible to understand molecular fundamentals of ontogenetic origins of cell variety in CNS [21–23].

In the given review, we will not delve into the technological aspect of the problem, but consider the main achievements of scRNA-seq/snRNA-seq technologies and spatial transcriptomics in SCT.

Literature search was conducted in PubMed database using the following key words: “spinal cord trauma”, “RNA sequencing”, “spatial transcriptomics”.

## Spinal neurons

Spinal neurons are characterized by an intensively marked heterogeneity. A clear understanding has been formed about cytogenetic, structural, cytochemical, and functional characteristics of motor neurons, interneurons, propriospinal, cholinergic, exciting, and inhibitory neurons [24–35]. However, as the results of the recent works with scRNA-seq/snRNA-seq have shown, all these populations appeared to be still more heterogenic in terms of differential gene expression [15, 17, 36–38]. Information on heterogeneity of spinal neuron populations is of great applicational importance for identification of neurons more or less susceptible to damage (disease resistance) in order to find the targets in traumatic injury and neurodegenerative diseases, for example, in lateral amyotrophic sclerosis [14, 39–41].

The results of the spinal cord study engaging the RNA-seq technology enabled the researches to establish the decisive role of neural network topography in the organization of the spinal cord of mammals. Thus, using scRNA-seq, the main differences between dorsal and ventral neurons were defined on the criteria of cluster formation and differential gene expression controlling neuroplasticity [17]. This observation is critically important for understanding the differences in the regenerative potential of the neurons of specific populations in pathologic conditions. The dorsal clusters differ in distinctly divided concrete neuron types, which are easily grouped into families. These neurons are located at a large distance from each other and may be reliably discernable. On the contrary, ventral clusters of neurons are closer to each other, with close or overlapping distribution in the tissue and overlapping patterns of gene expression. These differences in the structural and molecular organization of the dorsal and ventral parts of the spinal grey matter may underlie the specificity of neuron network functioning in these regions. Higher plasticity of connections in the dorsal area may be due to reactions such as central sensitization, progressive enhancement of nociceptive neuron excitability, long-term potentiation, depression, which accompany chronic neuropathic pain. In the ventral part, connections are structurally more stable, which is evidenced, in particular, by a high gene expression coding the synthesis of perineuronal net components stabilizing synaptic connections and limiting neuron plasticity [17].

The scRNA-seq data are crucially important in cell biology since they allow one to reconceive the boundaries of phenotype and classical definition of the cell belonging to the specific type as the cells with identical set of genes allowed for expression regardless whether they are transcribed or not.

In the study by Blum et al. [37], 43,890 transcriptomes have been profiled in the material of the mature mouse spinal cord, enriched with nuclei of efferent neurons, and a detailed characteristic of gene expression was given at the level of a separate cell. Efferent neurons of the spinal cord make up only 0.4% of all cells in the organ. Therefore, to profile transcriptomes in the snRNA-seq technology, fluorescence-activated sorting and enrichment of nuclei were performed. Cholinergic neurons were presented by 20 clusters. Also, there were identified 16 clusters of sympathetic neurons, which differed in localization and expression of the genes of neuromodulator signaling including several clusters localized in the sacral part of the spinal cord. Here, autonomic neurons of various subtypes express different combinations of neuromodulating peptides such as somatostatin, neurotensin, and proenkephalin. This study allowed for identification of new genetic markers specific for autonomic and somatic motor neurons, for α- and γ-motor neurons, and also establishment of heterogeneity of γ-motor neurons, whose various types express different transcriptional programs [37].

Differences in gene modules have been characterized in detail in electrophysiologically and metabolically various populations of fast and slow α-motor neurons. These types of motor neurons differ in the set of potassium channel subunits, which control resting potentials and excitation speed [24]. Marked transcriptional heterogeneity of somatic motor neurons correlates with electrophysiological characteristics and localization of the motor pools [37].

Heterogeneity of cell populations formed after traumatic spinal cord injury in the lumbar region of mice was comprehensively defined by means of snRNA-seq [15]. In this study, 17,354 nuclei have been sequenced, seven main clusters detected and presented by the following cell types identified by expression of the markers: 52% of neurons, 16% of oligodendrocytes, 14% of a mixed population of meningeal and Schwann cells, 9% of astrocytes, 5% of vascular cells, 1% of oligodendrocyte precursors, and 1% of microglia. Forty-three populations of neurons, which were unevenly distributed across the clusters in different regions of grey matter, have been identified and molecularly described. Thus, 55% of neurons were concentrated in 25 dorsal clusters, 34% — in 13 ventral clusters, and 11% of neurons were localized in 5 clusters — in dorsal horns and in the intermediate zone. The dorsal part of grey matter contained the cell population, which differed mainly in gene expression, whereas the ventral segment demonstrated the overlapping patterns of gene expression [15].

Motor neurons of the lateral motor nucleus are present in the cervical and lumbar parts of the spinal cord and control extremity muscle contraction, whereas motor neurons of the medial motor nucleus are distributed across the entire rostro-caudal axis of the organ and are connected with the axial musculature. In vertebrates, molecular identity of motor neurons of the mentioned nuclei is generally known. Nevertheless, the identity of subtypes in these cell populations innervating separate groups of muscles remained unclear. The answer to this question was received using snRNA-seq [38]. The motor neurons of the medial motor nucleus are subdivided into three subtypes, which are distinguished by the expression pattern of genes *Satb2*, *Nr2f2*, and *Bcl11b* and depend on the localization of the neurons along the mediolateral axis and expression of molecules controlling the directed axon growth.

When establishing therapeutic targets, identified molecular diversity allows for tracing and comparing susceptibility and regenerative potential of different subtypes of neurons in the models of CNS damage and neurodegenerative diseases, for example, lateral amyotrophic sclerosis [40, 42]. The scRNA-seq/ snRNA-seq technologies have become actively used to analyze molecular and cellular mechanisms of pathologic reactions and regeneration in SCT [11, 43–50].

To find the causes of selective cell susceptibility in CNS pathology, it is important to have detailed information on the specificity of human cell population and compare them to the known data obtained on the experimental models. Using snRNA-seq and spatial transcriptomics, 29 clusters of glia and 35 neuron clusters [51], arranged mainly by the anatomical principle, have been identified. The information resource created on these data is of vital importance for a clinical picture. Spinal motor neurons, which degenerate in lateral amyotrophic sclerosis and other neurodegenerative diseases relative to other spinal neurons have been shown to express genes controlling the cell size and cytoskeleton structure, which suggests the availability of specialized molecular repertoire underlying their selective susceptibility.

Interesting results obtained by Sun et al. [52] are worth mentioning to illustrate the clinical significance of the data on RNA sequencing of the spinal cord cells in mice with the models of neurodegenerative diseases. In this study, the most prominent shifts were found not in the neural cells of the mice with the model of spinal muscular atrophy, as it was expected, but in the subpopulation of vascular fibroblasts [52]. The number of cells in this subpopulation decreased essentially, which led to the vascular defects with the following inhibition of energy metabolism and protein synthesis.

A lumbar spine represents a special interest for the analysis of posttraumatic reactions and is considered as an important therapeutic target regardless of the level of spinal cord injury [53, 54]. It is primarily connected with the presence of interneuron networks in this part forming a central generator of patterns and controlling the motor function [55, 56]. In case of SCT in the lumbar spine, rearrangement of nervous connection occurs alongside with the reorganization of the descending motor pathways [57–59]. Involvement of specific interneurons forming these networks, their belonging to the definite classes of precursors, molecular specificity, and capability of modulating a phenotype in response to the damage remain unclear. The scRNA-seq/snRNA-seq technologies were recognized to be the most informative in solving the complex of these issues in SCT.

Application of snRNA-seq technology made it possible to acquire essentially new data on the mechanisms of regeneration of the motor function in severe contusion SCT in a mouse in response to epidural electrostimulation [48]. The SCT in the middle thoracic segment caused complete disintegration of corticospinal tract fibers and a marked reduction of the amount of glutomatergic fibers of the reticulospinal tract more caudally from the injury region. In the lumbar segment of the spinal cord, of 82,093 nuclei subject to the transcriptome analysis, 20,990 belonged to the neurons which were assigned to 36 populations. Electrostimulation resulted in immediate activation of exciting interneurons localized in the intermediate laminae of the lumbar spinal segment, which expressed *Vsx2* gene (Visual System Homeobox 2) and the marker of the caudal spine neurons *Hoxa10* (Homeobox A10). Kathe et al. [48] managed to establish in the process of elegant experiments that these neurons were not involved in the control of stepping in the intact spinal cord but were triggered in the regeneration of walking after SCT. The authors believe that a positive effect of epidural electrostimulation on the restoration of the motor function in the clinical picture of 9 patients with SCT might be associated with the activation of exactly these interneurons.

The population of neurons (~21%) prevails in the intact lumbar spinal cord of a mouse. On the model of severe contusion SCT in the thoracic region of the mouse spinal cord, no significant changes in the ratio of populations of exciting, inhibiting, and motor neurons (8:8:1) were registered in the lumbar spinal cord segment by means of snRNA-seq [49]. Meanwhile, signs of enhancement of synaptic plasticity and sensitivity to the action of neurotransmitters are noted in this part of the spinal cord, as well as activation of the genes encoding synthesis of their receptors, synaptogenesis, and remodeling of synapses.

In case of SCT in the thoracic spinal cord segment, neurons of the lumbar region remain generally save in contrast to the neurons in the epicenter of the damage [60, 61]. However, already in a week after the SCT, the lumbar spinal neurons demonstrate shifts in the expression of the genes encoding the cell stress molecules including redox reactions and protein folding, and also the molecules of neurotransmitter-mediated signaling and ion channel functioning. At the same time, some populations of exciting neurons in the dorsal horns and inhibiting neurons in the ventral horns are characterized by alterations in the organization of synapses and gene expression connected with plasticity [49]. After SCT, physiological cascades in the majority of the lumbar neurons are inhibited and the genes connected with neurotransmission and restructuring of synapses are activated. Unlike these reactions, regeneration-associated genes begin to express in the neurons of two concrete populations, namely, in Shox2-expressing V2d and in the neurons of the spinocerebellar tract after the injury. The data obtained with RNA sequencing give reason to believe that neurons of the lumbar networks belonging to different populations may demonstrate specific strategies of restoration [49].

In SCT, transplantation of neural precursors into the spinal cord stimulates regeneration of axons of corticospinal tract and recovery of the motor function [46, 62]. In order to define molecular mechanisms of such an action, the regeneration transcriptome (reversion to the embryonic state) of motor neurons localized in the layer V of the motor cortex, the axons of which form a corticospinal tract, have been analyzed [46]. Only SCT, as well as the trauma in combination with transplantation of neural precursors, cause similar early transcriptome responses in motor neurons. Two weeks after SCT, this transcriptome is inhibited, but when the trauma is combined with cell transplantation, it appears more stable. Huntingtin gene (*Htt*) is a central gene-concentrator (hub) in the regeneration transcriptome including regeneration-associated genes and programs [63–67]. *Htt* deletion significantly weakens regeneration, which indicates a key role of this gene in neuron plasticity after trauma [46].

## Astroglia

Heterogeneity of the astrocyte population is generally recognized [68–71]. The scRNA-seq technology widens essentially our notion in this field, allows us to receive new data on the astrocyte population in the spinal cord and their behavior in SCT [72]. Glial fibrillary acid protein (GFAP) is considered the most reliable marker for identification of astrocytes. The scRNA-seq technology made it possible to identify the populations of GFAP-expressing cells in intact, sham-operated, and traumatized mice with spinal cord compression in the caudal thoracic region. Populations of astrocytes different in the expression of gene including those controlling proliferative activity, were defined in the acute and chronic stages. Astrocytes expressing the markers specific only for this cell types and astrocytes, which along with their markers expressed the markers of ependymal cells, were also identified. The amount of cells with a mixed expression significantly increased in the acute phase of SCT, they were localized at a distance from the central channel both in the intact and traumatized mice. These astrocyte-ependymal cells were present both in white and grey matter, but their quantity in white matter prevailed [72]. The increase in such cells in the acute phase has been also unexpectedly found in the sham-operated animals (with laminectomy only). This observation raises the question of an adequacy of using animals of this group as a control in SCT.

The molecular profile, change in the duration of the cell cycle phases, similar to the radial glia morphology of astrocyte-ependymal cells with high expression of nestin, the marker of neural stem cells [73], not only confirm the data on the origin of one of the astrocyte populations from the neural stem cell, localized in the ependymal layer of the spinal cord, but indicate to the presence of similar cells in the intact spinal cord [72]. Despite the capability of differentiation into astrocytes [74] and oligodendrocytes, ependymal stem cells in SCT seem to be more predisposed to the generation of astrocytes, which make up about half of the total number of astrocytes of the glial scar [75].

## Oligodendroglia

Mature oligodendrocytes also show transcription heterogeneity, functional consequences of which are not clear. Their heterogeneity may correlate with the influence of microenvironment or interaction with various neuron types. Some populations of oligodendrocytes in the mammalian CNS have been shown to possess spatial arrangement [44]. Oligodendrocytes type 2 prevail in the spinal cord, whereas oligodendrocytes type 5 and 6 increase their contribution to the oligodendrocyte lineage with age in all analyzed regions of CNS. Oligodendrocytes type 2 and 5/6 differ in the presence in the motor and sensory tracts. Progenitors of oligodendrocytes in neurogenesis seem not to be specified for differentiation into the cells of these populations. Reactivity of oligodendrocytes type 2 and 5/6 is different in chronic SCT. Oligodendrocytes type 2 decrease their contribution into the oligodendrocyte population in the damaged region and increase it in the areas of nerve fiber degeneration, especially in the chronic phase of SCT [44]. Increased presence of oligodendrocytes types 5/6 in the general population in the site of injury indicates that factors, which stimulate resident oligodendrocyte progenitors to preferential differentiation into oligodendrocytes types 5/6, are active in this region. On the whole, scRNA-seq in complex with immunofluorescence analysis show that various populations of oligodendrocytes differ in spatial preferences, differently react to the SCT, and may perform different functions in the course of regeneration.

Two clusters of oligodendrocyte progenitors, A and B, have been identified in acute SCT in the thoracic region using scRNA-seq. Cells with the phenotype A predominate. These cells express classical genes for oligodendrocyte progenitors encoding synthesis of the molecules such as chondroitin sulfate proteoglycan 4 (CSPG4, or NG2-proteoglycan), receptor of platelet-derived growth factor α (PDGFRα), and tenascin R. Oligodendrocyte progenitors B appear on the first day after injury and express actively tenascin C [47]. Oligodendrocyte progenitors A are supposed to be involved in the processes of cytogenesis and myelination, while the cells of population B actively proliferate [47].

## Ependymal glia

Neural stem cells in the formed spinal cord are present in the population of the ependymal cells. Their multiplication is not registered in the human spinal cord [76, 77], although *in vitro* ependymocytes enter mitosis [78–80]. Ependymal glia became the object of experimental studies at the level of a separate cell [45, 81, 82]. In a number of works, data have been obtained on the manifestation of a neural stem cell potential by some population of ependymocytes [82, 83]. Ependymocytes and neural stem cells in the adult organism originate from the common embryonal progenitors [82, 84]. A comprehensive analysis of population heterogeneity and age-related ependymocyte transcriptome in the spinal cord was undertaken in the study [50]. In the general population of ependymocytes, scRNA-seq allowed for identification of immature cells as potential stem cells in the spinal cord. After the trauma, these cells enter again the cell cycle, which is accompanied by a short-term reversion of their maturation.

Resident neural stem cells make a limited contribution to cell replacement. In traumatic injury of the mouse spinal cord, ependymal cells give rise mainly to astrocytes of the glial scar [75, 82] and to a lesser extent to oligodendrocytes [85–87]. A latent potential of the resident neural stem cells for replacing a significant part of the dead oligodendrocytes in the injured mouse spinal cord has been detected. The scRNA-seq technology demonstrates neural stem cells being in the permissive state, which allows realization of the usually latent program of gene expression for oligodendrogenesis after the injury.

In the formed spinal cord, the oligodendrocyte marker, transcription factor Olig2, does not cause a stimulating effect on oligodendrogenesis and myelination, but early progenitors in the ependymocyte population preserve the possibility of the response to the action of this transcription factor, and this is observed in the case of SCT. Ectopic Olig2 expression accompanies intensive oligodendrogenesis, generated from the stem cells, which follows the natural differentiation of oligodendrocytes, promotes remyelination of axons, and stimulates restoration of nerve fiber conductivity [45]. These data give the opportunity to suppose that recruiting resident stem cells may serve as an alternative to cell transplantation after CNS damage.

## Microglia

Microglia is presented by the resident immune cells in CNS, which participate in the immune defense, maintenance of homeostasis, phagocytosis of the dead cell fragments, pruning of excess synapses and axon collaterals, growth stimulation, and remyelination of neuron projections [88–91]. Presently, the scRNA-seq/ snRNA-seq technologies are widely used to study the diversity of microglia [47, 92–95]. RNA sequencing has identified new microglia markers: S100A8, S100A9, HEXB, TMEM119, GPR34, P2RY12, Siglec-H, TREM2, OLFML3 [96].

A separate and difficult task, which was successfully and completely solved owing to the RNA-sequencing technology, consisted in differentiating microglia from macrophages. These cell lineages are characterized by morphological identity and similar phenotypic markers *in vivo*, especially in pathology including SCT, when both cell types are activated [95, 97–100].

Recent investigations using scRNA-seq have established that microglia differs from the border-associated macrophages by expression of the genes encoding purinergic receptor P2yr, membrane transporter SLC2A5 (solute carrier family 2 member 5), transmembrane protein Tmem119, and β-subunit of β-hexosaminidase (Hexb), whereas border-associated macrophages express the molecule of lymphocyte activation Ms4a7 (member 7 subfamily 4 of the membrane-spanning domain), mannose receptor (Mrc1), and others [95]. These data are important for identifying and studying the role of border-associated macrophages in CNS, which control the delivery of leucocytes from blood and cerebrospinal fluid to the brain parenchyma, and also limit CNS and blood exchange with different cytokines and chemokines [101].

In SCT, microglia coordinate specifically the interaction of different cell types [102]. Pharmacological depletion of microglia aggravates spinal cord injury and worsens function recovery. On the contrary, restoration of intracellular signaling cascades in microgliocytes, identified by the scRNA-seq data, prevents the secondary damage and facilitates regeneration. The analysis of this work [102] shows that optimal recovery after SCT may be achieved through coordination of the key ligand-receptor interaction between microglia, astrocytes, and infiltrating leukocytes.

A spinal cord trauma triggers neuroinflammatory reaction, in which monocytes/macrophages and resident microglia cell predominate. The scRNA-seq technology distinguishes homeostatic and non-homeostatic microglia in the spinal cord. In SCT, non-homeostatic microglia include three populations, namely, inflammatory, proliferating, and migrating microglia. The inflammatory microglia are characterized by the expression of genes associated with cell death, cytokine production, and expression of the purinergic *P2ry12* receptor gene, which weakly expresses in two other populations of non-homeostatic microglia. The proliferating microglia express genes associated with regulation of the cell cycle, for example *Cdk1*. Microgliocytes of the smallest population of migrating cells express cell mobility-related genes and are characterized by high levels of expression of macrophage scavenger receptor gene (*Msr1*) and insulin-like growth factor 1 gene (*Igf1*) [47].

In SCT, spatial transcriptomics reveals phenotypes of monocytes, macrophages of several subtypes, namely those inducing chemotaxis and proinflammatory ones, and border-associated macrophages and dendrite cells in the clusters of myeloid cells [47]. Besides, the mentioned macrophage subtypes do not comply with the well-known classification of polarization phenotypes M1/M2. Inducing chemotaxis and proinflammatory macrophages demonstrated patterns of interaction similar to those in astrocytes and fibroblasts. These intercellular signaling-related data, obtained by means of scRNA-seq, contribute to the understanding of the molecular mechanisms of astrogliosis, fibrosis, and angiogenesis in SCT.

The spinal cord trauma is followed by many reactions of different cell types, which are specific in time, by the terms, and location. In mice traumas, application of scRNA-seq in combination with the traditional analysis of the structure, behavior, and electrical activity made it possible to obtain data on temporal and molecular shifts at the level of a single cell. There were described pathological changes in the cells of 12 main types, of them three types of cells migrated to the spinal cord at different time after trauma [103]. In the intact spinal cord, new subtypes of microglia have been detected with individual dynamic transformations typical for each of them and specific for various stages of the pathological process. Activation of microglia occurs by two waves. The most marked microglia changes are noted on days 3 and 14 after SCT. In the subacute period, when manifestations of neuroinflammation are most dynamic, the microglia reaction is followed by the activation of seven gene-concentrators (hub genes) *Itgb1*, *Ptprc*, *Cd63*, *Lgals3*, *Vav1*, *Shc1*, and *Casp4* [104]. By day 38, the main cell types are still deviated significantly from the state in the intact spinal cord [103].

The majority cell types in a traumatized spinal cord return, as a rule, to the initial state, whereas the possibility of constant reprogramming of the molecular profile leading to a prolong change of the immune status in the traumatized spinal cord is considered for microglia. In SCT of mice, microgliocytes with increased expression of regeneration-associated molecules have been detected. Similar cells are typical for newborn mice but with small differences in gene expression relative to their neonatal analogs [103].

## Conclusion

The choice of a therapy mode in the traumatic spinal cord injury requires an understanding of the role of the key genes and the respective intracellular regulatory pathways. However, the selection of single genes for this purpose does not allow one to disclose deep molecular mechanisms of the spinal trauma pathogenesis. The constructive solution of this task began with the application of the scRNA-seq/snRNA-seq technologies.

The results of recent investigations using scRNA-seq/snRNA-seq have shown that all earlier known populations of spinal neurons appeared heterogeneous according to the criterion of differential gene expression. This information has acquired an important clinical significance for identification of the most susceptible neurons in traumatic spinal cord injury. The RNA-sequencing technologies helped define molecular-genetic correlates of functional differences and regenerative potential of neurons localized in the dorsal and ventral laminae, which may be the basis of specific functioning of neural networks in these regions of grey matter. Activation of interneurons was found in the lumbar spinal cord region in response to the remote trauma in the thoracic spine. These interneurons are not involved in the stepping act in the intact spinal cord but are activated after trauma and the following epidural electrostimulation. The RNA-seq data give reason to suppose that the neurons, pertaining to various populations, forming motor networks, may realize different molecular programs of regeneration.

The scRNA-seq/snRNA-seq technologies expand our notion about heterogeneity of spinal astrocytes in traumatic spinal cord injury. They differ in expression of genes controlling proliferative activity. Astrocytes, expressing markers of the ependymal cells, were identified both in white and grey matter. The number of these astrocytes increases significantly in the acute phase of the spinal cord trauma.

Molecular profile, change in phase duration of the cellular cycle, similar to the radial glia morphology of the astrocyto-ependymal cells with a high expression of nestin, the marker of stem cells, not only confirm the data on the origin of one of the astrocyte population from the stem neural cell localized in the ependymal spinal cord layer but point to the presence of the similar cells in the intact spinal cord.

The scRNA-seq data in complex with the immunofluorescence analysis show that spinal oligodendrocytes of various populations are topographically different, differently react to the SCT, and may fulfil diverse functions in the process of regeneration.

The RNA-seq technologies made it possible to establish and analyze in detail heterogeneity of the population and age dynamics of ependymocyte transcriptome in the spinal cord. Data were obtained on the manifestation of the neural stem cell potential in ependymocytes of some subpopulation and unfolding a normally latent program for oligodendragenesis after spinal cord trauma. These technologies enabled obtaining new data on saving by the early precursors in the ependymocyte population the possibility to respond to the action of transcription factor Olig2, a stimulator of oligodendrogenesis.

The scRNA-seq/snRNA-seq technologies have revealed new microglia markers, by which activated microglia and macrophages can be reliably discriminated; enabled receiving new data on involvement of microglia in the specific mechanisms of coordinating the interaction between the different types of spinal cells in traumatic injuries of the spinal cord. The scRNA-seq technology provided the opportunity to divide microglia into homeostatic and non-homeostatic and to distinguish in the latter the subpopulations of inflammatory, proliferating, and migrating microglia.

In traumatic spinal cord injury, spatial transcriptomics describes monocyte phenotypes inducing chemotaxis in the clusters of myeloid cells; proinflammatory and border-associated macrophages, and dendrite cells. The data obtained assume the possibility of constant reprograming of the glial molecular profile leading to a prolonged change of the immune status in the traumatized spinal cord.

The development of RNA-sequencing technologies and spatial transcriptomics opens great opportunities for formulation of novel promising conceptions concerning the mechanisms of neural connection reorganization and restoration of sensorimotor functions after the spinal cord trauma.
